# Sexual side effects of antipsychotic drugs in schizophrenia: Protocol for a systematic review with single-arm, pairwise and network meta-analysis of randomized controlled trials and non-randomized studies

**DOI:** 10.12688/f1000research.154742.1

**Published:** 2024-08-28

**Authors:** Johannes Schneider-Thoma, Shimeng Dong, Orestis Efthimiou, Spyridon Siafis, Wulf Peter Hansen, Elfriede Scheuring, Karl Heinz Möhrmann, Stefan Leucht

**Affiliations:** 1 TUM School of Medicine and Health, Klinikum rechts der Isar, Department of Psychiatry and Psychotherapy, Technical University of Munich, Munich, Germany; 2 Partner Site München/Augsburg, German Center for Mental Health (DZPG), Munich, Germany; 3 Institute of Primary Health Care (BIHAM), University of Bern, Bern, Switzerland; 4 BASTA-Bündnis für psychisch erkrankte Menschen, Munich, Germany; 5 Landesverband Bayern der Angehörigen psychisch erkrankter Menschen e.V., Munich, Germany

**Keywords:** Sexual side effects, antipsychotics, schizophrenia, meta-analysis

## Abstract

**Introduction:**

Sexual dysfunctions are common yet underreported side effects of antipsychotics for schizophrenia, affecting 30-80% of treated individuals. These side effects can severely impact social interactions and treatment adherence for individuals with schizophrenia, but comprehensive comparative evidence assessing the risk profiles of different antipsychotics is lacking. This study aims to address this gap using network meta-analysis that integrates data from both randomized-controlled trials (RCTs) and non-randomized studies (NRS).

Protocol

This systematic review will include both RCTs and NRS focusing on participants with schizophrenia or schizophrenia-like psychoses, without restrictions on symptoms, gender, ethnicity, age, or setting. For interventions, all second-generation antipsychotics will be included. The primary outcome will be the occurrence of at least one sexual adverse event of any kind. Secondary outcomes will be the occurrence of any sexual adverse event evaluated in men and women separately, and any adverse event related to the three phases of sexual response cycle separately: desire (e.g. libido, sexual thoughts), arousal (e.g. erection, lubrication) and orgasm (e.g. ejaculation, anorgasmia), and any adverse effect related to breast dysfunction and menstruation irregularities. Study selection and data extraction will be performed independently by two reviewers. The Cochrane Risk of Bias tool 1 and ROBINS-I will be employed to evaluate the risk of bias for RCTs and NRS, respectively. Single-arm meta-analysis of proportions will synthesize the average frequency of sexual adverse events in treated participants. Pairwise and network meta-analysis of RCTs and NRS will be used to evaluate comparative tolerability. Subgroup and sensitivity analyses will explore possible heterogeneity in results and validate the findings’ robustness. The quality of the evidence will be evaluated using GRADE.

**Discussion:**

This study will provide vital insights into the sexual side effects of antipsychotics by combining evidence from clinical trials and real-world practice, facilitating better decision-making in choosing the optimal antipsychotic for individuals.

## Introduction

Schizophrenia is a prevalent severe mental illness with worldwide distribution, affecting approximately 1% of the population during their lifetime due to its start during early adulthood ( McGrath et al. 2008). Antipsychotics, which are critical for both acute management and prevention of relapse in schizophrenia ( DGPPN e.V. for the Guideline Group 2019), are often prescribed over long periods, potentially lifelong. These medications, however, are associated with various side effects, including sexual dysfunctions.

Sexual dysfunctions induced by antipsychotics can manifest as disturbances in sexual desire, erection and ejaculation, vaginal lubrication, and orgasmic dysfunctions as well as partly related disorders of the menstruation cycle and the breast (such as gynecomastia and galactorrhea) ( Kelly and Conley 2004; La Torre et al. 2013; Montejo et al. 2018). These dysfunctions are not only common —mostly reported in 30-80% of treated individuals with prevalence rates varying from 0 to over 90% ( La Torre et al. 2013) —but also highly distressing and a frequent cause of non-adherence to treatment ( Perkins 2002; Lambert et al. 2004). Non-adherence significantly elevates the risk of relapse of psychotic symptoms. Moreover, sexual side effects critically interfere with normal participation in social life in terms of having close and satisfying personal relations in a romantic partnership, which is one of the most important unmet needs of individuals with schizophrenia ( Jager and McCann 2017). Therefore, sexual side effects severely diminish the quality of life for those affected ( Bebbington et al. 2009; Olfson et al. 2005).

Despite the significant clinical impact of sexual side effects induced by antipsychotics, there is a lack of comprehensive meta-analyses addressing this critical issue, particularly no network meta-analyses presenting differences between antipsychotics in this regard. Existing reviews include several narrative reviews and some pairwise meta-analyses (mainly Cochrane reviews) that only focused on specific antipsychotics and invested sexual side effects as secondary outcomes (risperidone ( Hunter et al. 2003; Jayaram and Hosalli 2005; Komossa et al. 2011), sertindole ( Komossa et al. 2009; Lewis et al. 2005), paliperidone ( Harrington and English 2010), or amisulpride ( Men et al. 2018)). Moreover, some meta-analyses only included observational studies ( Zhao et al. 2020; Korchia et al. 2023) or had a small number of studies ( Trinchieri et al. 2021). One single-arm meta-analysis combined both randomized and observational data and calculated overall percentages of sexual dysfunctions with each antipsychotic across 34 studies ( Serretti and Chiesa 2011). However, as the authors report themselves, this approach is not suitable to make statements for differences between antipsychotics in propensity to cause sexual side effects. In summary, the existing evidence leaves us with an incomplete and only impressionistic picture which is limited in terms of available trials, number of events and use of inappropriate methods.

This study aims to fill this knowledge gap by providing evidence-based insights on sexual adverse events associated with antipsychotic to guide the selection of the optimal drug for individual needs. Therefore, to summarize according to the PICO(S) scheme, we will conduct a comprehensive network meta-analysis combining data from randomized-controlled trials and real-world observational studies ( **S**tudy design) to compare all second-generation antipsychotics ( **I**ntervention) with each other ( **C**omparator) on their propensity to cause sexual side effects ( **O**utcome) in patients with schizophrenia ( **P**opulation).

## Methods

We report this systematic review and network meta-analysis protocol according to the Preferred Reporting Items for Systematic review and Meta-analysis Protocols (PRISMA-P) checklist, and the PRISMA extension for network meta-analysis ( Hutton et al. 2015). The PRISMA-P Checklist can be found in the extended data. This protocol has been registered with PROSPERO (registration number: CRD42024510190) and will be updated with any necessary amendments.

### Criteria for considering studies for this review

Study designs

We will include randomized controlled trials (RCTs) and non-randomized studies (NRS). RCTs identified with high risk of bias in sequence generation will be considered as quasi-randomized studies and grouped with NRS. The inclusion of NRS is not limited to specific study designs because as stressed by the Cochrane handbook ( Reeves et al. 2022), design labels are used very inconsistently and the risk of bias of a certain NRS can be only assessed when the specific study features are known. Accordingly, studies will first be classified by design, followed by a careful assessment of bias risk for each study and studies with critical risk of bias will be excluded from the analysis. We will also exclude studies from mainland China that are not conducted by international pharmaceutical companies or published in international scientific journals due to significant concerns regarding methodological and reporting quality ( Leucht et al. 2022). Both open-label and blinded studies will be included; however, open-label and single-blind studies will be excluded in a sensitivity analysis to address potential bias in expectations of sexual side effects. The minimum study duration will be 3 weeks because shorter studies usually do not focus on clinical efficacy and tolerability of antipsychotics but on more experimental research questions. For cross-over studies, only data from the first phase will be used to avoid carry-over effects, which are common in schizophrenia.

Participants

We will include trials in which at least 80% of the participants are diagnosed with schizophrenia or related disorders (such as schizophreniform or schizoaffective disorders) without restrictions in terms of symptoms (acute episode or maintenance phase), gender, ethnicity, age, or setting. These inclusion criteria are adopted because occurrence of side effects can be considered largely independent of psychopathology and they will increase the data availability for these typically underreported outcomes ( Zorzela et al. 2016). Of note, we will record potentially important population characteristics for each trial and consider them in the assessments of heterogeneity and transitivity ae well as in subgroup and sensitivity analyses.

Interventions

All second-generation antipsychotics (SGAs), which are predominantly prescribed for schizophrenia in Europe, Japan and the USA, will be included in this study, namely amisulpride, aripiprazole, asenapine, blonanserine, brexpiprazole, cariprazine, clozapine, iloperidone, lumateperone, lurasidone, olanzapine, olanzapine-samidorphan, paliperidone, perospirone, quetiapine, risperidone, sertindole, ziprasidone, zotepine. Only SGAs are included because those were investigated in recent clinical research adhering to standardized procedures. These standards include systematic documentation of adverse events according to protocols like the Good-Clinical-Practice guideline and use of standardized nomenclatures of adverse events such as MedDRA ( International Council for Harmonisation of Technical Requirements for Pharmaceuticals for Human Use 2022). Furthermore, reporting of studies involving SGAs typically comply with guidelines like CONSORT for RCTs ( Schulz et al. 2010) and STROBE for NRS ( Vandenbroucke et al. 2007), ensuring detailed information on study design and outcomes. Moreover, study authors and pharmaceutical companies of these trials are very likely to keep electronic records and are contactable to provide necessary additional information, which is very important for this review. However, we will include first-generation antipsychotics (FGAs), placebo and no treatment when they were used as comparators in RCTs and NRS of SGAs.

We will include all these compounds, when used in monotherapy, in any form of administration (e.g. oral or intramuscular depot). Primarily, different applications of the same drug will be combined because side effects predominantly follow the pharmacodynamic profile of the specific compounds and not its pharmacokinetics, as observed in previous reviews ( Huhn et al. 2019; Schneider-Thoma et al. 2022), but considered separate interventions in sensitivity analysis. For RCTs, we will only include fixed-dose studies within the target to maximum range according to a recent consensus reached after a two-step Delphi survey among international experts in the treatment of schizophrenia ( McAdam et al. 2023); all flexible-dose treatment regimens (as long as they overlap with the target to maximum range) will be included as these allow investigators to titrate doses to optimal levels for individual participants. Similarly, NRS that rely on observed clinical data will be treated as having flexible dose. In sensitivity analyses, we will exclude flexible dose RCTs and NRS in which the applied doses were outside the target to maximum range for some participants, to control for potential effects of extremely low or high doses.

Comparators

In network meta-analysis there is no formal comparator as all interventions will be compared with each other.

Outcome measures

*Primary outcome*

The primary outcome will be “Any sexual side effect”. We will use the occurrence of at least one sexual adverse event of any kind provided by the original authors, for example from specific questionnaires for sexual side effects. In case the occurrence of any sexual side effect is not explicitly reported, we will use the highest number of participants reported for any specific sexual adverse event, in line with methodologies used in previous reviews ( Serretti and Chiesa 2011; Huhn et al. 2019; Schneider-Thoma et al. 2022).

*Secondary outcomes*

1. Any sexual adverse event in men and women separately.
2. Any adverse events related to the “desire” phase of sexual response cycle, such as libido decrease, loss of sexual thoughts.
3. Any adverse events related to the “arousal” phase of sexual response cycle, such as erectile dysfunction, vaginal lubrication decrease.
4. Any adverse events related to the “orgasm” phase of sexual response cycle, such as ejaculation dysfunction, anorgasmia.
5. Any adverse related to breast dysfunction, such as gynecomastia, galactorrhea.
6. Any adverse related to menstruation irregularities, such as amenorrhea.

Of note, there is discussion whether breast dysfunction and menstruation irregularities should be considered as sexual side effects because they are not part of the sexual function per se. However, they are frequently mentioned in parallel to dysfunctions of the sexual response cycle, included in some scales for sexual side effects ( Serretti and Chiesa 2011; Boer et al. 2014) and very bothersome for participants, and therefore we decided to address them as secondary outcomes.

Timing

Timing of outcome measurement will be at study endpoint.

### Search strategy

Electronic searches

As recommended by the PRISMA harms checklist ( Zorzela et al. 2016) and the Cochrane handbook ( Reeves et al. 2022), we search for any study that might have reported adverse events in general and not only for studies mentioning specific sexual adverse events in title/abstract because it is impossible to report all adverse events in searchable/indexable parts of publications. For RCTs, we search the Cochrane Schizophrenia Group’s Study-Based Register of trials ( Shokraneh and Adams 2020) for published and unpublished reports. Following the methods from Cochrane ( Higgins JPT, Thomas J, Chandler J, Cumpston M, Li T, Page MJ, Welch VA 2022) the Information Specialist compiles this register from systematic searches in MEDLINE, Embase, Allied and Complementary Medicine (AMED), Cumulative Index to Nursing and Allied Health Literature (CINAHL), PsycINFO, PubMed, US National Institute of Health Ongoing Trials Register ClinicalTrials.gov, World Health Organization International Clinical Trials Registry Platform ( www.who.int/ictrp), ProQuest Dissertations and Theses A&I. The register also includes hand searches and conference proceedings and does not place any limitations on language, date, document type or publication status. For NRS, we search multiple electronic databases including ClinicalTrials.gov, Embase, MEDLINE, PsycINFO, Science Citation Index-Expanded, and WHO International Clinical Trials Registry Platform (ICTRP) with no date/time, language, document type, and publication status limitations. The search string contains terms for schizophrenia and the included antipsychotics. The detailed search strategies can be found in the extended data.

Reference lists and other sources

As additional hand searches, we will check the included studies in previously published relevant systematic reviews. Moreover, because adverse events are often underreported, we will contact the corresponding authors of each included study for unpublished information about adverse events.

### Identification and selection of studies

Using Rayyan ( Ouzzani et al. 2016), title and abstracts of identified references are screened in duplicate by two reviewers with regard to the eligibility criteria above. Any disagreements between the two reviewers are solved by discussion. Then, again in duplicate, two reviewers will inspect the full articles of references selected in title/abstract screening for eligibility and for availability of sexual side effects. Any disagreements will be solved by discussion among the two reviewers or with a third, experienced reviewer (JST, SL). If a decision cannot be made, the study authors will be contacted for clarification.

### Data extraction

Two reviewers will independently conduct data extraction in an established server-based Microsoft Access database designed for the specific needs of blind data entry by two reviewers and automatic double check of extracted data. Data extraction will be piloted on a random sample of ten RCTs and ten NRS. In case of disagreement, a decision will be reached by discussing with a third reviewer (JST, SL) or by contacting the study authors. Data on the following points will be collected:

− General information, such as author name, year of publication, treatment arms and sample size.
− Methodology, such as study design, blinding, duration of study, diagnostic criteria used, study population (Intention-to-treat, observed cases) for which adverse events are reported.
− Participant characteristics, such as age, weight, number of men/women, diagnosis details, plasma prolactin level.
− Intervention characteristics, such as doses, form of application, percent co-medication with antidepressants.
− Outcome measures.

### Risk-of-bias assessment

Risk of bias will be assessed for each included study by two reviewers in duplicate referring to the Cochrane Collaboration’s risk of bias tools for randomized controlled studies (RoB tool 1) and non-randomized studies (Risk Of Bias In Non-randomized Studies – of Interventions, ROBINS-I). Disagreements in the assessment will be discussed among the two reviewers and, if needed with a third, experienced reviewer (JST, SL). We will exclude NRS judged as carrying an overall critical risk of bias from the primary analysis. RCTs judged at high risk of bias RCTs and NRS judged at serious risk of bias, we will exclude in a sensitivity analysis.

### Data analysis

**Overview of the step-wise process for data synthesis of randomized and non-randomized data**

First, we will conduct frequentist random effects single-arm and pairwise meta-analyses with RCTs and NRS as subgroups to synthesize estimates of overall prevalence and comparative tolerability and to assess heterogeneity. In the next step, we will conduct network meta-analysis of RCTs (including assessment of transitivity and evaluation of consistency). If the network meta-analysis of RCTs is internally consistent, we proceed with comparing the different estimates from RCTs (direct, indirect, mixed evidence) to the estimates of NRS. If there are no indications for systematic differences between RCT and NRS estimates, we proceed with combined network meta-analysis (again including assessment of transitivity and inconsistency).

Of note, if the requirements for network meta-analysis of RCTs or joint network meta-analysis of RCTs and NRS are not met, we will not proceed to the next step and use pairwise meta-analysis or network meta-analysis of RCTs for data synthesis.

**Details of synthesis**

*Estimation procedures*

For estimating the proportion of patients experiencing side effects in antipsychotics, we will use the number of participants experiencing sexual adverse events and non-events among pa exposed to antipsychotics or placebo/no treatment. We will meta-analyze the data using generalized linear mixed models ( Schwarzer et al. 2019).

For comparative pairwise and network meta-analysis, the number of participants experiencing sexual adverse events will be synthesized using odds ratios (OR) because ORs have better mathematical properties for meta-analysis, particularly in the case of studies with varying prevalence rates ( Doi et al. 2022) and because it is the only measure available in case-control studies. If available in the original publication, we will use reported ORs that are adjusted for possible confounders, such as differences in age and sex between the compared groups. If not available, we will calculate ORs based on the number of participants with events and the number of participants assessed (considering that some sexual adverse events only occur in men or women).

For the pairwise meta-analysis we aim to use a random effects meta-analysis model. However, if the data are sparse, i.e. if there are many studies with few or zero events in one or more of their arms, the usual inverse variance model for meta-analysis has limitations. In that case we will use models that can better handle rare events, such as the Mantel-Haenszel model and Bayesian approaches, as per methodological recommendations ( Efthimiou 2018).

Network meta-analysis of RCTs will be performed in a frequentist framework using a random effects model. We will assume a common heterogeneity parameter across the various treatment comparisons. For combined network meta-analysis of RCTs and NRS, different several statistical models are available. The selection of the most suitable model will be decided after careful consideration of the actual data, the distribution of studies by designs and the risk of bias assessment ( Efthimiou et al. 2017). In case of sparse data, we will explore the use of a Bayesian model or a frequentist model based on the Mantel-Haenszel approach ( Efthimiou et al. 2019).

*Assessment of heterogeneity*

Heterogeneity (variability in relative treatment effects within the same treatment comparison) will be assessed within and across study designs by visual inspection of forest plots and estimating the statistical heterogeneity τ, i.e. the standard deviation of random effects, and I ^2^. We will employ empirical distributions to characterize the amount of heterogeneity as low, moderate or high ( Turner et al. 2012). Substantial heterogeneity indicates important differences in clinical and methodological characteristics of the studies which warrant further investigation, such as checking for mistakes in data entry and for potential effect modifiers and bias factors. Moreover, to assess how much heterogeneity affects the clinical interpretation of the relative treatment effects with respect to the extra uncertainty anticipated in a future study, we will produce prediction intervals.

*Assessment of the transitivity assumption in network meta-analysis*

Joint analysis of treatments can be misleading if the network is substantially intransitive. Intransitivity can arise when design, population or treatment characteristics that may modify the relative effects between interventions are distributed differently between comparisons. For the case of relative treatment effects in terms of sexual side effects, there is no clear a-priori-evidence, but several characteristics, may play a role (e.g. study design, blinding, gender, age, dose, antidepressant co-medication). Therefore, we will investigate if relevant characteristics are similarly distributed across studies grouped by comparison.

*Assessment of inconsistency*

Consistency, i.e. the agreement between direct evidence and indirect evidence of a network meta-analysis, will be statistically evaluated globally, by using the design-by-treatment test ( Higgins et al. 2012) and locally, via the back-calculation method ( König et al. 2013). In case of evidence of inconsistency, we will investigate possible sources of it (mistakes in data entry, clear differences in study characteristics).

*Investigation of heterogeneity and inconsistency*

Substantial heterogeneity, intransitivity or inconsistency will prevent network meta-analysis. Small or moderate amounts will be further explored by subgroup, network meta-regression, and sensitivity analyses.

We a priori plan to investigate the impact of following potential effect modifiers via Bayesian network meta-regression analyses of the primary outcome: percentage women, mean age, prolactin level, percent co-medication with antidepressants (which also cause sexual side effects), and duration of study. Additionally, we will perform separate network meta-analyses for sexual adverse events occurring in men and women (see secondary outcome).

Moreover, we will explore the robustness of results (with regard to the inclusion of studies with differences in study design, population and intervention characteristics in the primary analysis) by sensitivity analyses. The following sensitivity analyses of the primary outcomes are predefined: exclusion of (1) non-double-blind studies, (2) studies that report only observed-case analyses, (3) RCTs with high risk/NRS with serious risk of bias, (4) flexible-dose studies in which the range of applied doses exceeded the recommended (target to maximum) dose range ( McAdam et al. 2023), (5) studies that did not use specific questionnaires to assess sexual side effects, (6) studies in acutely ill patients (because acute psychosis might interfere with sexual functioning). Moreover, we will perform network meta-analysis with oral and depot applications of the same compound as separate interventions.

Small study effects and publication bias

For assessment of small study effects and publication bias, we will employ a comparison-adjusted funnel plot method to explore the association between study size and effect size ( Chaimani and Salanti 2012). Moreover, comparisons with 10 or more studies will be plotted in a contour-enhanced funnel plot ( Peters et al. 2008). Similarly, we will plot a contour enhanced funnel plot of all SGAs combined versus placebo.

Assessment of the confidence in estimates

The quality of the evidence of the primary outcome will be evaluated using the Grading of Recommendations Assessment, Development and Evaluation (GRADE) framework extended to NMA ( Puhan et al. 2014).

Statistical software

Analyses will be performed in R using the packages “meta” for single-arm and pairwise meta-analysis ( Balduzzi et al. 2019), “netmeta” for network meta-analyses ( Rücker et al. 2020), “crossnma” for combined network meta-analyses of RCTs and NRS ( Hamza and Salanti 2022). Bayesian analyses will be performed using self-programmed routines in “rjags” ( Plummer et al. 2023). R software and the mentioned packages are freely available https://cran.r-project.org/bin/windows/base/

Study status: search and the selection process are ongoing currently.

## Discussion

Despite its significant clinical relevance, there is a lack of scientific comparison between different antipsychotics regarding their sexual side effects. This project will address this gap by providing a comprehensive synthesis of evidence from clinical trials and real-world clinical practice. This information is relevant for clinicians and guideline developers in selecting the most appropriate medication for individuals. Additionally, this review is of high importance for future clinical research, regarding both RCTs and NRS, as it will report the current state of evidence concerning sexual side effects of antipsychotics and identify existing limitations. Finally, this review will be among the first to integrate randomized and non-randomized evidence in a network meta-analysis, thereby advancing methodological approaches in evidence-based medicine.

## Patients and public involvement

We collaborate with members of the patient organization “BASTA - Bündnis für psychisch erkrankte Menschen” and the relatives’ organization “Landesverband Bayern der Angehörigen psychisch erkrankter Menschen e.V.” in this project. They contributed in identifying the research idea and developing this review protocol from their perspective as people with lived experience with the disease schizophrenia and the treatment with antipsychotics. They will be updated regularly about the state of the project and help with any upcoming questions. Moreover, they will be involved in interpreting the results and in preparing a lay summary of the results so that other patients and relatives of patients can be directly informed about the scientific results with a text that can be understood, e.g. using the BASTA-newsblog ( http://www.bastagegenstigma.de/).

### Ethics and consent

This review does not require ethical approval.

### Contributions of authors

SL is the principal investigator, obtained funding, and supervises the study. JST, SD, SS and SL designed the study and provided clinical and methodological advice. JST and SD drafted the manuscript and registered the protocol with PROSPERO before. OE provided substantial methodological and statistical advice. WPH, ES and KHM provided the patient perspective when designing the study. All authors critically reviewed the manuscript for important intellectual content and approved its final version.

## Data Availability

No data associated with this article. Figshare: Sexual side effects of antipsychotic drugs in schizophrenia: Protocol for a systematic review with single-arm, pairwise and network meta-analysis of randomized controlled trials and non-randomized studies, https://doi.org/10.6084/m9.figshare.26396275.v2 ( Dong, 2024). The project contains following data:
• PRISMA-p checklist • Search strategy Data are available under the terms of the Creative Commons Attribution 4.0 International License (CC-BY 4.0).
