# Supplementary material for: Sexual dysfunction and other prolactin-related side effects of antipsychotic drugs in schizophrenia: Protocol for a systematic review with single-arm, pairwise, and network meta-analyses of randomized controlled trials and non-randomized studies
Source: F1000Res. 2025 Jun 19;13:973. Originally published 2024 Aug 28. [Version 3] doi: 10.12688/f1000research.154742.3 (PMC12138325; doi:10.12688/f1000research.154742.3)
Supplement: Peer review history

# Reviewer report: Reviewer response for version 3

**DOI:** 10.5256/f1000research.183803.r393283

**Published:** 2025-06-25

**Authors:** Angel L Montejo

**Affiliations:** 1 Hospital Universitario de Salamanca, University of Salamanca, Salamanca, Spain

No further comments.

Is the study design appropriate for the research question?

Partly

Is the rationale for, and objectives of, the study clearly described?

Yes

Are sufficient details of the methods provided to allow replication by others?

Partly

Are the datasets clearly presented in a useable and accessible format?

Yes

Reviewer Expertise:

Sexuality and Mental Health, Psychotropic-related sexual dysfunction, Pharmacology

I confirm that I have read this submission and believe that I have an appropriate level of expertise to confirm that it is of an acceptable scientific standard.

# Reviewer report: Reviewer response for version 2

**DOI:** 10.5256/f1000research.175947.r383765

**Published:** 2025-06-04

**Authors:** James MacCabe

**Affiliations:** 1 King's College London, London, England, UK

This is an important topic and this group are very skilled and experienced  in meta-analysis, particularly in the field of antipsychotics. The methods are clearly described and the authors have included the views of service users. I have no concerns and `I` link forward to reading the report in due course.

Is the study design appropriate for the research question?

Yes

Is the rationale for, and objectives of, the study clearly described?

Yes

Are sufficient details of the methods provided to allow replication by others?

Yes

Are the datasets clearly presented in a useable and accessible format?

Not applicable

Reviewer Expertise:

Treatment resistant pasychosis

I confirm that I have read this submission and believe that I have an appropriate level of expertise to confirm that it is of an acceptable scientific standard.

# Reviewer report: Reviewer response for version 2

**DOI:** 10.5256/f1000research.175947.r365735

**Published:** 2025-02-12

**Authors:** Angel L Montejo

**Affiliations:** 1 Hospital Universitario de Salamanca, University of Salamanca, Salamanca, Spain

The authors have addressed the concerns included in my peer review report appropriately.

Is the study design appropriate for the research question?

Partly

Is the rationale for, and objectives of, the study clearly described?

Yes

Are sufficient details of the methods provided to allow replication by others?

Partly

Are the datasets clearly presented in a useable and accessible format?

Yes

Reviewer Expertise:

Sexuality and Mental Health, Psychotropic-related sexual dysfunction, Pharmacology

I confirm that I have read this submission and believe that I have an appropriate level of expertise to confirm that it is of an acceptable scientific standard.

# Reviewer report: Reviewer response for version 1

**DOI:** 10.5256/f1000research.169806.r332301

**Published:** 2024-11-12

**Authors:** Angel L Montejo

**Affiliations:** 1 Hospital Universitario de Salamanca, University of Salamanca, Salamanca, Spain

It is a protocol devised by an expert team with extensive experience in this field. This is a clinical problem of great relevance in psychiatric practice and its strength lies in the fact that there is no comparative study among all the antipsychotics marketed. The results will undoubtedly be of great interest and will provide new information on the meta-analyses already carried out in this field.

 However, there are still important difficulties to avoid challenges in obtaining reliable information excluding some important confounding factors.

 The first is that clinical trials of antipsychotics almost never include validated questionnaires to measure sexual dysfunction and rely on spontaneous patient communications, which tends to underestimate the  frequency of sexual dysfunction. The second is that many studies on the subject do not consider only those patients who have previous sexual function versus those who are sexually inactive, which may contribute to underestimating the frequency of sexual dysfunction as well. The third is that the high frequency of comorbidity and the use of concomitant treatments seriously bias the results, especially with the use of serotonergic and euthymizing compounds such as lithium, carbamazepine and valproate, which are related to high frequencies of sexual dysfunction.

 It would be important for the authors to take these limitations into account to find the differences in actual clinical practice between antipsychotics.

 Finally, because gynecomastia, galactorrhea and amenorrhea are not in themselves part of sexual function but are adverse effects that require adequate attention, I think the title should be changed to another such as: Sexual and endocrinological side effects of antipsychotic drugs in schizophrenia.

Is the study design appropriate for the research question?

Partly

Is the rationale for, and objectives of, the study clearly described?

Yes

Are sufficient details of the methods provided to allow replication by others?

Partly

Are the datasets clearly presented in a useable and accessible format?

Yes

Reviewer Expertise:

Sexuality and Mental Health, Psychotropic-related sexual dysfunction, Pharmacology

I confirm that I have read this submission and believe that I have an appropriate level of expertise to confirm that it is of an acceptable scientific standard, however I have significant reservations, as outlined above.

# Response

**Published:** 2024-12-12

**Authors:** Shimeng Dong

**Affiliations:** Technical University of Munich, Munich, Bavaria, Germany

It is a protocol devised by an expert team with extensive experience in this field. This is a clinical problem of great relevance in psychiatric practice and its strength lies in the fact that there is no comparative study among all the antipsychotics marketed. The results will undoubtedly be of great interest and will provide new information on the meta-analyses already carried out in this field.

**Our response:** We thank the reviewer for the positive evaluation of our project.

 1) However, there are still important difficulties to avoid challenges in obtaining reliable information excluding some important confounding factors. The first is that clinical trials of antipsychotics almost never include validated questionnaires to measure sexual dysfunction and rely on spontaneous patient communications, which tends to underestimate the frequency of sexual dysfunction.

**Our response:** We thank the reviewer for the important point. As already indicated in our original protocol (page 9, “ *Investigation of heterogeneity and inconsistency*” section), we will ensure that a sensitivity analysis will be performed, for both our single-arm and network meta-analyses, using only the studies that used validated questionnaires for sexual side effects. Additionally, we will perform a subgroup analysis to compare the results from studies that used validated questionnaires to those that relied on spontaneous reporting, which will address the issue of underreporting. We would also like to note that absolute frequencies may be underestimated but the relative effects of different antipsychotics are less impacted and can be estimated with rare-event data.

 2) The second is that many studies on the subject do not consider only those patients who have previous sexual function versus those who are sexually inactive, which may contribute to underestimating the frequency of sexual dysfunction as well.

**Our response:** We thank the reviewer for the important point. We will make sure to record whether the study involved a subgroup of sexually active participants and ensure that an additional sensitivity analysis will be performed using only those participants with previous sexual function or who were sexually active at the time of enrollment. We have updated the protocol accordingly (page 9, “ *Investigation of heterogeneity and inconsistency*” section; please find below).

 “The following sensitivity analyses of the primary outcomes are predefined: exclusion of (1) non-double-blind studies, (2) studies that report only observed-case analyses, …, and (7) studies that did not specifically include participants with previous sexual function or who were sexually active at the time of enrollment.”

 3) The third is that the high frequency of comorbidity and the use of concomitant treatments seriously bias the results, especially with the use of serotonergic and euthymizing compounds such as lithium, carbamazepine and valproate, which are related to high frequencies of sexual dysfunction.

**Our response:** We thank the reviewer for the important point. We already indicated that we would examine the impact of antidepressant co-medication, but will also record other psychotropic drugs (e.g., lithium, carbamazepine, and valproate) and examine their impact in a Bayesian network meta-regression analysis. We have updated the protocol accordingly (page 9, “ *Investigation of heterogeneity and inconsistency*” section; please find below).

 “We a priori plan to investigate the impact of following potential effect modifiers via Bayesian network meta-regression analyses of the primary outcome: percentage women, mean age, prolactin level, percent concomitant psychotropic drugs that can also cause sexual side effects (e.g., antidepressants and mood stabilizers).”

 We would also like to note that we will examine the distribution of concomitant treatments across different antipsychotic groups, where an equal distribution will allow the estimation of relative effects that are not affected by concomitant treatments.

 4) Finally, because gynecomastia, galactorrhea and amenorrhea are not in themselves part of sexual function but are adverse effects that require adequate attention, I think the title should be changed to another such as: Sexual and endocrinological side effects of antipsychotic drugs in schizophrenia.

**Our response:** We thank the reviewer for the suggestion. Adding “endocrinological” side effects also implies that prolactin levels and other endocrinological parameters (such as insulin) have been investigated as key outcomes; however, these are not our outcomes of interest. We are only interested in prolactin levels for the examination of their association with sexual dysfunction, and such data will be collected only when a study reports both sexual side effects and prolactin levels (or hyperprolactinemia). Rather than “endocrinological” side effects, we propose that “Sexual dysfunction and other prolactin-related side effects of antipsychotic drugs in schizophrenia” may better suit our scope because they directly refer to gynecomastia, galactorrhea, and amenorrhea, without referring to prolactin per se. If the reviewer agrees, we will update the title accordingly at the stage of publication of our results.

# Reviewer report: Reviewer response for version 1

**DOI:** 10.5256/f1000research.169806.r319278

**Published:** 2024-09-20

**Authors:** Sofia Brissos

**Affiliations:** 1 Centro Hospitalar Psiquiátrico de Lisboa, Lisboa, Portugal

The authors focus on an important aspect of schizophrenia patients' treatment, but again, they will focus on data that has been previously collected, which may not be sufficient. For instance, the majority of these clinical trials have a small number of female patients, and many real-life patients have been excluded due to the "rigorous" inclusion criteria. Nevertheless, I am happy to see that the authors will conduct independent analysis in men and women separately. In that sense, although the authors plan to include real-world observational studies, we will have access to the reported side-effects of those studies only.

 Finally, I myself do not find of great interest a manuscript on what will be done; I think what is of interest to the reader is the result of what the authors are planning to analyze, and not just their intentions.

Is the study design appropriate for the research question?

Partly

Is the rationale for, and objectives of, the study clearly described?

Yes

Are sufficient details of the methods provided to allow replication by others?

Yes

Are the datasets clearly presented in a useable and accessible format?

Not applicable

Reviewer Expertise:

Schizophrenia, psychosis, forensic psychiatry

I confirm that I have read this submission and believe that I have an appropriate level of expertise to confirm that it is of an acceptable scientific standard, however I have significant reservations, as outlined above.

# Response

**Published:** 2024-12-12

**Authors:** Shimeng Dong

**Affiliations:** Technical University of Munich, Munich, Bavaria, Germany

1) The authors focus on an important aspect of schizophrenia patients' treatment, but again, they will focus on data that has been previously collected, which may not be sufficient. For instance, the majority of these clinical trials have a small number of female patients, and many real-life patients have been excluded due to the "rigorous" inclusion criteria. Nevertheless, I am happy to see that the authors will conduct independent analysis in men and women separately. In that sense, although the authors plan to include real-world observational studies, we will have access to the reported side-effects of those studies only.

**Our response:** We thank the reviewer for the important point. We acknowledge that RCTs are highly selective in terms of recruiting participants and even non-randomized studies may not truly reflect the real-world setting. However, there are also advantages to be recognized because RCTs allow the estimation of the effect directly coming from a single antipsychotic, for instance, by restricting the use of concomitant drugs. Non-randomized studies usually do not make such restrictions, and whether the effect is truly caused by the antipsychotic becomes less clear, but at the same time allowing what the effect might be in real-life patients. Therefore, it is of high importance to examine both randomized and non-randomized studies, make comparisons, and provide more comprehensive evidence. We agree with the reviewer that although we plan to include observational studies, we will have access to the reported side-effects of those studies only. We will address this limitation by contacting the authors of the studies that do not necessarily report sexual side effects but potentially have them recorded (e.g., studies that only report common side effects, such as > 5% incidence).

 2) Finally, I myself do not find of great interest a manuscript on what will be done; I think what is of interest to the reader is the result of what the authors are planning to analyze, and not just their intentions.

**Our response:** We understand that protocols may not be as interesting to read as published results, but it is important for systematic reviews to have protocols before their implementation and make them public so that bias can be reduced and readers can see whether what was originally planned was actually done. We will of course publish the results later.
